# The Genus *Ravenelia*: Insights on Taxonomy, Diversity and Distribution

**DOI:** 10.3390/pathogens13090775

**Published:** 2024-09-09

**Authors:** Shubhi Avasthi, Ajay Kumar Gautam, Rajnish Kumar Verma, Kunhiraman C. Rajeshkumar, Mekala Niranjan, Amita Sharma, Samantha Chandranath Karunarathna, Nakarin Suwannarach

**Affiliations:** 1 School of Studies in Botany, Jiwaji University, Gwalior 474011, Madhya Pradesh, India; 2 Patanjali Herbal Research Department, Patanjali Research Institute, Haridwar 249405, Uttarakhand, India; 3 Department of Plant Pathology, Punjab Agricultural University, Ludhiana 141004, Punjab, India; vermarajnish1985@gmail.com; 4 National Fungal Culture Collection of India (NFCCI), Biodiversity and Palaeobiology (Fungi) Group, MACS Agharkar Research Institute, Pune 411004, Maharashtra, India; rajeshfungi@gmail.com; 5 Dharmavana Centre of Excellence for Biodiversity & Climate Resilience, Hyderabad, Secunderabad 500051, Telangana, India; scientist.niranjan@dharmavananatureark.org; 6 Department of Botany, Kurukshetra University, Kurukshetra 136119, Haryana, India; amita.sharma22@kuk.ac.in; 7 Center for Yunnan Plateau Biological Resources Protection and Utilization, College of Biological Resource and Food Engineering, Qujing Normal University, Qujing 655011, China; samanthakarunarathna@gmail.com; 8 Center of Excellence in Microbial Diversity and Sustainable Utilization, Chiang Mai University, Chiang Mai 50200, Thailand; suwan.462@gmail.com; 9 Department of Biology, Faculty of Science, Chiang Mai University, Chiang Mai 50200, Thailand

**Keywords:** current status, ITS and LSU, phylogeny, *Pucciniales*, rust fungi, taxonomy

## Abstract

*Ravenelia* is the third largest rust genus of the order *Pucciniales* with more than 200 described species. It is an important rust genus that has undergone tremendous taxonomic changes. This genus produces teliospores united into a head on a compound pedicel composed of two to several hyphae with autoecious, macro-, demi- to hemi-, and, more rarely, microcyclic modes of their life cycle which provide it a unique identity and have proved helpful in the identification of the genus. The current understanding of the taxonomy, history, diversity and distribution of this genus is discussed in this paper. Both online and offline resources were searched to gather data of the published literature. The data thus obtained were analyzed for numerical and graphical summaries to provide the diversity and distribution of the genus. In addition, a phylogenetic analysis based on the ITS and nLSU DNA sequence data available in GenBank and the published literature was performed to examine the taxonomic placement of different species within the genus. The genus was reported to be distributed over 53 countries of the world. Around 51 plant genera belonging to four plant families, viz., *Fabaceae*, *Phyllanthaceae*, *Asphodelaceae* and *Zygophyllaceae* were found to be infected with these rust fungi. The phylogenetic analysis based on LSU and ITS sequence data revealed the polyphyletic nature of the genus. A table of 248 species of this genus is also provided with all information of host, distribution and cited reference that can be helpful for mycologists to find all information at one place. Future perspectives for the advancement of this genus are also discussed.

## 1. Introduction

The genus *Ravenelia* is one of the largest genera of the order *Pucciniales* with more than 200 species described and found worldwide [1]. The species of this genus are tropical and subtropical biotrophic in nature and have a broad geographical distribution around the globe. It parasitizes the trees and bushes of families *Fabaceae* and *Euphorbiaceae* with high host specificity towards *Mimosoideae, Faboideae* and *Caesalpinioideae* (*Fabaceae*). This genus produces teliospores united into a head on a compound pedicel that provides them a unique identity and proved helpful in the identifying of the genus [2]. The wide distribution of the genus can be depicted by the fact that over 100 species have been reported from America, around 33 species from India, more than 40 species from Africa and about 20 species have been reported from East and Southeast Asia [3,4,5]. Several species of this genus are reported from neotropics, mostly from Mexico, Brazil and Argentina. However, their occurrence in other countries, such as Panama and Costa Rica, is poorly studied [6,7]. There are only 18 species that have been reported from Costa Rica while a single species (*R. entadae*) was from Panama [8]. Most East and Southeast Asian species were reported from China and Taiwan [9,10].

The genus was introduced in the year 1853 by Berkeley; over time, the genus has undergone several taxonomic and systematic transformations. However, all species of *Ravenelia* shared the most prominent morphological features including the production of multicellular teliospores on compound pedicels composed of two to several hyphae with autoecious and macro- and demi- to hemi-, and, more rarely, to microcyclic modes of their life cycle [1,11]. With the addition of many species in recent years, this genus has become one of the largest genera of rust fungi [1]. Some previous researchers used morphological features or host information to define the species. In contrast, others grouped multiple taxa under a single name that reflected a species complex by emphasizing morphological similarities [12]. Based on morphological characters and life cycles, mycologists in the early twentieth century established sections or even split this genus into several distinct genera [2,13,14,15,16].

The present compilation presents a comprehensive review that includes a detailed taxonomic description of the genus *Ravenelia*. A complete history of the genus depicted from its establishment to the current scenario is also discussed. A global overview of the diversity and distribution of the genus *Ravenelia* is also presented along with their currently accepted species, geographical diversity and distribution and phylogenetic position and sexual morphs are provided. Updated phylogenetic analysis for the whole genus based on multigene DNA sequence data available in different databases like GenBank and the published literature is also performed to examine the intergeneric relationships of the genus *Ravenelia*. In addition to a broad taxonomical description, a brief life cycle, ecological significance and other comparative aspects of its diversity and distribution are also provided.

## 2. The Genus *Ravenelia*: Historical Perspective

The genus was proposed by Berkeley [17] and later monographed by Dietel [14,18]. As per the present scenario, around 250 species have been accepted as valid [1,7]. There were two species, namely, *R. glandulosa* Berk. & M.A. Curtis and *R. indica* Berk. which were initially included in the genus at the time of introduction. The *R. glandulosa* was transferred from *Sphaeria epiphylla* Schwein. which was earlier collected on *Tephrosia virginiana* (L.) Pers. in South Carolina [19,20]. Similarly, *R. indica* was observed on the leaves of *Abrus* sp. from Jharkhand, India [17]. However, Dietel correctly recombined the type species to *R. epiphylla* (Schwein.) [18]. However, the species *R. glandulosa* has been declared Nom. inval., Art. 36.1(c) (Melbourne) and transferred to the current name as *R. glanduliformis* Berk. & M.A. Curtis [21,22]. Many new species have been added to the genus over time and it became the third-largest rust genus after *Puccinia* and *Uromyces* [1,2,10,23]. In the twentieth century, many mycologists established sections or even split this genus into several distinct genera. Sydow [16] distinguished eight genera based on teliospore characteristics and life cycles. Different nomenclatural systems were proposed for the genus *Ravenelia* based on the attributes of spore stages like aeciospores (I) and teliospores (III). The nomenclatural systems proposed by Long [13] included *Ravenelia*, *Pleoravenelia* and *Neoravenelia* with Peridia and Caeoma aeciospores and 1-/2-layered teliospores. Similarly, Dietel [14] proposed three sections, namely sects. *Pleo*-/*Haploravenelia*, sect. *Pleoravenelia* and sect. *Haploravenelia* for the genus *Ravenelia* based on Peridia. Peridia + Caeoma aeciospores and 1-/2-layered teliospores. In both nomenclatural systems, any life cycle mode can be found. In the nomenclature proposed by Sydow and Sydow [15] two genera were included: *Ravenelia* and *Neoravenelia* with the Peridia and Caeoma aeciospores as well as 1-/2-layered teliospores. In a fourth proposed nomenclature by Sydow [16], genera like *Ravenelia*, *Haploravenelia*, *Neoravenelia*, *Longia*, *Cystotelium*, *Dendroecia*, *Cephalotelium* and *Cystingophora* were included. Here the aeciospores (Peridia and Caeoma, 1-/2-layered) and teliospores (1-/2-layered and Peridia + Caeoma) were also analyzed and hemicycle, macrocyclic and demicyclic modes of life cycle were presented in this system. However, a broad genus concept that includes all these proposed genera or divisions (within one genus) is still considered the most frequently recognized genus concept of *Ravenelia.* Buller [24] investigated the homothallic and heterothallic nature of rust fungi. He observed that the microcyclic forms of rust fungi were heterothallic because they produced fully functional pycnia, whereas other species were homothallic. However, the pycnia of the genus *Ravenelia* were found intermediate between well-developed pycnia of heterothallic species and vestigial pycnia of homothallic ones. Later, Hiremath and Pavgi [25] studied dikaryotization in some species of *Ravenelia*. In their experiment on two species of *Ravenelia,* i.e., *R. breyniae* Sydow on *Melanthesa rhamnoides* Blume and *R. sessilis* Berkeley on *Albizzia lebbek* Benth., they found both species to be homothallic. They also concluded that dikaryotization is affected by cellular fusion in *R. breyniae* and by nuclear division in *R. taslitnii* Mundkur. Earlier studies depicted the different morphological, cytological and physiological characteristics of *Ravenelia*. Singh [26,27] studied detailed morphology, cytology and physiology of various species of *Ravenelia*, whereas, Hiremath and Pavgi [28], Pavgi and Singh [29] and Pavgi and Hiremath [30] performed their investigations on pycnia. They observed a total of three types of pycnia (intraepidermal, subepidermal and subcuticular from *R. breyniae*, *R. emblicae* and *R. taslimii* respectively) in their study. Similarly, a study on the morphology of spore forms of *R. hobsoni* Cke. also revealed the presence of pycnia, uredia and telia in its life cycle [31,32].

Long [33] prepared a note on new and rare species of *Ravenelia* and presented it. The author considered characteristics such as the position of sori (sub-epidermal or sub-cuticular) as well as the number and position of germ pores and cysts in that study. Singh [34,35] studied hyperparasitism on different species of *Ravenelia* and identified *Cephalosporium coccorum* Petch as a mycoparasite on uredia and telia of *R. breyniae* and *R. hobsoni* and *Colletotrichum gloeosporioides* Penzig. on *R. sessilis*. Rezende and Dianese [36] carried out a detailed investigation on native leguminous hosts for the association of phytopathogenic fungi from the Brazilian Cerrado. The study results revealed four new species of *Ravenelia viz. R. cerradensis* on *Chamaecrista clausenii* var. *cyclophylla*, *R. chapadensis* on *Chamaecriista conferta* var. *virgate*, *R. mineirosensis* on *Anadenanthera colubrina* var. *colubrina* and *R. emaensis* on *Anadenanthera* sp. A novel gall-inducing acacia rust species of the genus *Ravenelia* along with new host associations in Hawaii was studied by Scholler and Aime [37] and in South Africa by Ebinghaus et al. [3]. These fungi were identified based on molecular phylogenetic analyses of DNA sequence data (LSU and ITS rDNA regions) and morphological examinations. Similarly, two more species, namely, *R. piepenbringiae* and *R. hernandezii*, were described based on morphology and molecular phylogenetic analyses (28S rDNA sequence data) from the Neotropics, Panama and Costa Rica [38]. A study carried out in Thailand on newly found *Ravenelia* species with their additional host and life cycle stage reported the occurrence of *R. odoratissimae, R. ornate, R. parasnathii* and *R. tandonii* on *Albizia odoratissima*; on *Abrus pulchellus, Ab. precatorius*, and *Abrus* sp.; on *Acacia comosa* and an unidentified *Acacia* species; and on *Acacia catechu*, respectively. *Ravenelia odoratissimae* was found to produce a uredinial stage in addition to a telial stage in its life cycle [10]. Another study in South Africa rediscovered six *Ravenelia* species, five of which represented new reports for South Africa [2]. This ongoing research on fungal biodiversity exploration, resulting in the discovery of new species and records, raised the number of species within the genus, rendering this the third-largest genus of rust fungi.

## 3. Taxonomic Treatment

The presence of multicellular teliospores, borne on compound pedicels composed of two to several hyphae, is a major characteristic commonly shared by all the species of *Ravenelia*. All the *Ravenelia* species are autoecious with macro-, demi-, hemi- or microcyclic life cycles, and produce five spore states, i.e., spermatia (0), aeciospores (I), urediniospores (II), teliospores (III) and basidiospores (IV). The disease symptoms produced by *Ravenelia* species on their respective hosts include the appearance of ochraceous to light brown and chestnut to dark brown pustules on the abaxial and adaxial leaf surfaces. The production of galls and witches’ brooms in infected host tissues during the aecial stage and uredinoid aecia in many *Ravenelia* spp. are important features of these rust fungi [1,11].

*Ravenelia* Berk. Gard. Chron., London 13: 132 (1853)
*= Cystingophora* Arthur, North American Flora 7 (2): 131 (1907)
*= Haploravenelia* Syd., Annales Mycologici 19 (3-4): 165 (1921)
*= Neoravenelia* Long, Botanical Gazette Crawfordsville 35: 131 (1903)
*= Pleoravenelia* Long, Botanical Gazette Crawfordsville 35: 127 (1903)
*= Cystotelium* Syd., Annales Mycologici 19 (3-4): 165 (1921)
*= Dendroecia* Arthur, Resultats Scientifiques du Congres International de Botanique Vienne 1905: 340 (1906)
*= Longia* Zeller, Mycologia 35 (4): 414 (1943)
*= Cistingophora* Arthur (1907)
*Classification*: *Raveneliaceae, Pucciniales, Pucciniomycetes, Pucciniomycotina, Basidiomycota*
*Type species*: *Ravenelia glanduliformis* Berk. & M.A. Curtis, Grevillea

**Figure 1:**
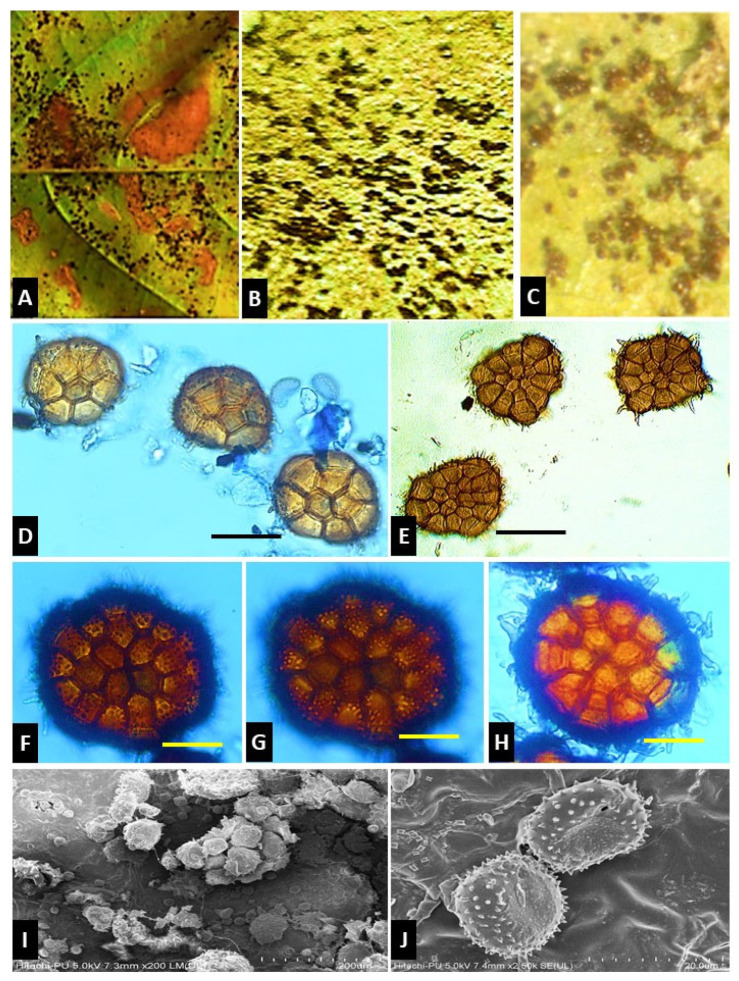
*Ravenelia* sp. on *Pongamia* sp. (**A**–**C**) Rust sori on infected leaf, (**D**–**H**) teliospore of *Ravenelia* sp., (**I**,**J**) SEM images of teliospore of *Ravenelia* sp. (Scale bar: **D**–**J** = 20 µm).

The multicellular teliospores are borne on compound pedicels composed of two to several hyphae. These spores have an ellipsoidal, reniform or almost hemispherical shape in lateral view and bear various pendent hygroscopic cysts. Spermogonia are mostly subcuticular, group VI (type 5), however, they are subepidermal in a few cases with type 7 spermogonia [1]. However, these are usually absent. Aecia are subepidermal (subcuticular also) and erumpent, containing pedicellate spores (*Uredo* type) or catenulate spores in some cases with or without peridium (*Aecidium-* or *Caeoma-* type). Uredinia are of *Uredo*-type, mostly subepidermal (subcuticular and erumpent), containing echinulate urediniospores borne singly on pedicels. Telia are mostly abaxial, subepidermal (sometimes subcuticular) and dark brown to nearly black in color. The multicellular, ellipsoidal, reniform or almost hemispherical shaped teliospores consisting of 1-cell-thick (2-cells-thick in some species) discoid heads are borne on compound pedicels composed of two to several hyphae. There is a single germ pore present in each cell of teliospores. This genus contains external basidia. Some *Ravenelia* species have dikaryotic haustoria and intracellular hyphae [1,2] (Figure 1).

**Figure 2:**
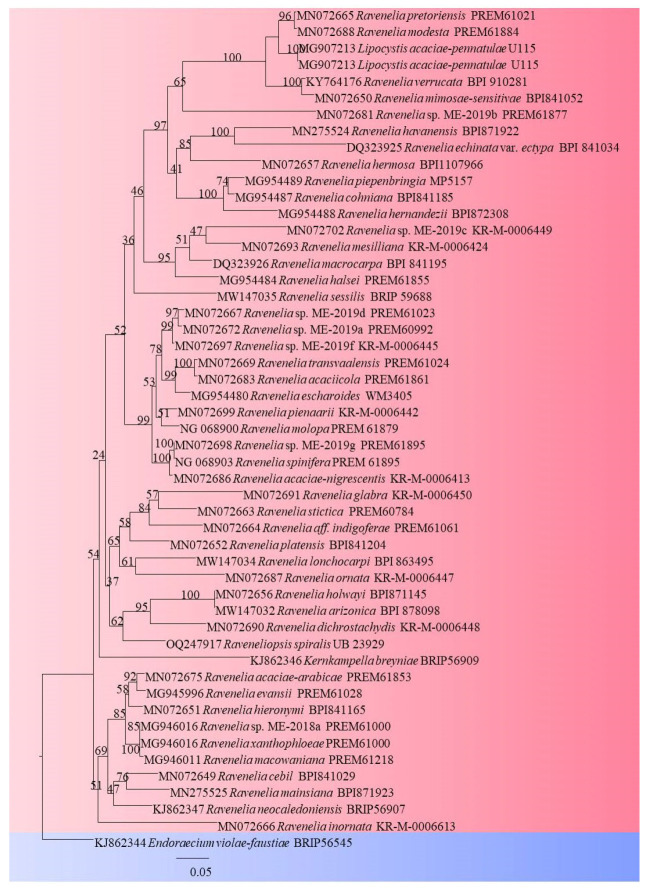
Phylogenetic tree generated by maximum likelihood analysis of combined LSU sequence data of *Ravenelia* species including *Endoraecium violae-faustiae* as an outgroup taxon. ML bootstrap values ≥ 70% are shown respectively. Fifty-one sequences are included in the phylogenetic analyses with 1169 characters including gaps. The best RaxML tree with a final likelihood value of −11,289.310130 is presented. The maximum likelihood dataset consisted of 666 distinct alignment patterns, with 17.89% of undetermined characters or gaps. Estimated base frequencies were as follows: A = 0.298160, C = 0.159641, G = 0.261176 and T = 0.281023; substitution rates AC = 1.210064, AG = 3.865525, AT = 1.571968, CG = 0.567293, CT = 5.233400 and GT = 1.000000; proportion of invariable sites I = 0.373594; and gamma distribution shape parameter α = 0.434322.

Although morpho-taxonomic traits account for the identification of species of *Ravenelia*, few species are represented by molecular data. The 18S and 28S regions of nuclear rDNA extracted from herbarium specimens or dried materials and field collections of rust fungi including *Ravenelia* were examined in a study on rust fungi (*Pucciniales*) to provide a better systematic outline of rust fungi [39]. The phylogenetic position of *Ravenelia esculenta* was evaluated based on the 18S rDNA region by Gandhe and Kuvalekar [40]. As per the literature, this was the first report of DNA sequencing and phylogenetic placement of the genus *Ravenelia* from India. A rust disease of *Vachellia* sp. was investigated in South Africa and pathogenic fungi were identified as *R. macowaniana* and *R. evansii* based on DNA sequence data of LSU and ITS rDNA regions analyses along with morphological characteristics [3]. The phylogenetic analyses based on 28S rDNA sequence data revealed the identity of *R. piepenbringiae* and *R. hernandezii* as a causal organism of rust of *Senegalia* plants in Panama and Costa Rica [38]. A study on *Ravenelia* spp. with a special focus on South Africa resulted in six novel *Ravenelia* species. Along with morpho-taxonomy, 28S rDNA and Cytochrome-c-oxidase subunit 3 was also used in molecular phylogenetic analyses of *Ravenelia* spp. [2]. A stable higher-rank classification of rust fungi was proposed by Aime and McTaggart [23] based on the phylogenetic analysis of multigene sequences from type species or type species proxies. Three DNA gene loci, namely nuclear large subunit, small subunit rDNA and Cytochrome-c-oxidase subunit 3, were used in this phylogenetic study that resulted in a higher-rank classification or taxonomic framework and evolutionary and diversification trends within *Pucciniales* [23]. A taxonomic framework of Indian *Pucciniales* was outlined by Gautam et al. [5] and included only five species of *Ravenelia* for phylogenetic analysis based on ITS and LSU regions of rDNA. Notably, out of 33 *Ravenelia* spp. reported from India, only a few have molecular data. Many rust fungi, including *Ravenelia* spp., still lack molecular data that may point out their uncertain taxonomic placement. Currently, a total of 321 epithets of rust genus *Ravenelia* have been recorded on various plant hosts [21]; many lack molecular data and need to be recollected in order to generate molecular data for correct taxonomic placement. It was inferred from the published literature that gene loci like Cytochrome oxidase subunit 3 (*CO3*), Large subunit ribosomal RNA, Small subunit ribosomal RNA, Internal Transcribed Spacer (ITS) and beta-tubulin (*B-tub1*) gene are some of the gene loci that were used in all molecular studies carried out earlier [2,5,23,41]. Different gene loci utilized in the present study during the molecular analysis of *Ravenelia* are given in Table 1 and Figure 2.

## 4. Diversity and Distribution

*Ravenelia* is the third largest genus after *Puccinia* and *Uromyces* in *Pucciniales*. The genus is global in distribution and species are reported to infect different plants. With the discovery of type species, more than 250 described species are widely distributed in subtropical and tropical regions. Based on the careful examination of published literature, it was found that the majority of *Ravenelia* spp. were reported on the host plant family *Fabaceae* (97.98%), whereas few species were also found on other families like *Phyllanthaceae* (0.80%), *Asphodelaceae* (0.40%) and *Zygophyllaceae* (0.40%). More than 51 host plants were reported to be infected with these rust fungi, whereas species of *Acacia* exhibited highest specificity to different species of *Ravenelia.* The global host range of *Ravenelia* species depicted the occurrence of this rust fungi on 51 genera of different plant families. The different plant hosts reported to be infected by species of *Ravenelia* are as follows: *Abrus, Acacia, Albizia, Aloe, Anadenanthera, Andira, Brogniartia, Bulnesia, Caesalpinia, Calliandra, Cassia, Cassiaria, Cenostigma, Chamaecrista, Cracca, Cratylia, Dichrostachys, Elephantorrhiza, Entada, Enterolobium, Erythrina, Gleditsia, Hoffmannseggia, Indigofera, Igna, Leucaena, Leucania, Lonchocarpus, Lysiloma, Megonemium, Millettia, Mimosa, Mundulea, Parkia, Phyllanthus, Piptadenia, Piscidia, Pithecellobium, Plathymenia, Poinciana, Pongamia, Prosopis, Pseudopiptadenia, Senegalia, Senna, Sesbania, Siderocarpos, Sophora, Swartzia, Tephrosia* and *Vachellia* (Table 2).

**Figure 3:**
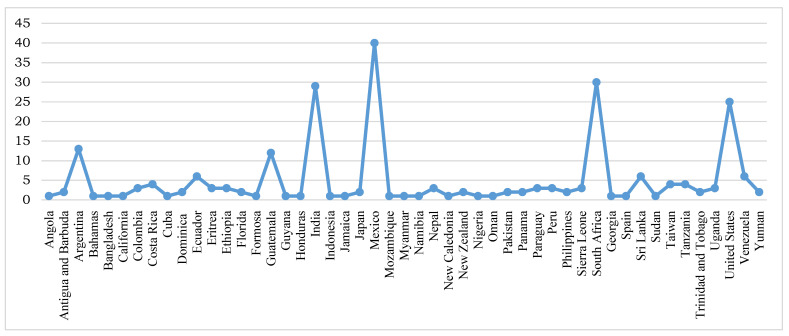
Country-wise distribution of *Ravenelia* species across the world.

Distribution: Angola, Antigua and Barbuda, Argentina, Bahamas, Bangladesh, Brazil, Chile, Colombia, Costa Rica, Cuba, Dominica, Ecuador, Eritrea, Ethiopia, Florida, Formosa, Guatemala, Guyana, Honduras, India, Indonesia, Jamaica, Japan, Mexico, Mozambique, Myanmar, Namibia, Nepal, New Caledonia, New Zealand, Nigeria, Oman, Pakistan, Panama, Paraguay, Peru, Philippines, Sierra Leone, South Africa, Georgia, Spain, Sri Lanka, Sudan, Taiwan, Tanzania, Trinidad and Tobago, Uganda, United States, Venezuela, Yunnan, Guatemala, Argentina. Based on the literature retrieval information, maximum reports on the occurrence of *Ravenelia* spp. were observed in countries like Mexico, South Africa, India and the United States (Figure 3 and Table 2).

## 5. Statistics and Discussion

**Figure 4:**
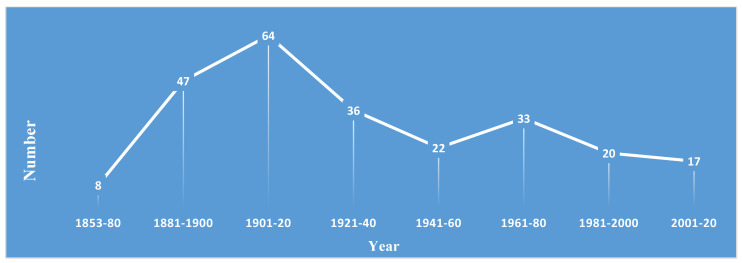
Number and trend of *Ravenelia* species introduced from 1853 to 2020 (based on data retrieved from Index Fungorum).

When we analyzed the publication of studies pertaining to *Ravenelia* spp., an increasing trend has been observed from the year of establishment of the genus (1853) to the year 2000. Afterward, this trend was observed in an undulating manner, i.e., it was observed increasing and decreasing. The data collected from the published literature on the genus *Ravenalia* depicted that more than 150 published papers described the reports on new species/taxon. After assessing the trend on the number of fungal names over 20 years, variable results were observed. Initially, the number of fungal names continued to increase from the publication of the first species of fungi in 1853 to 1920. A slight decrease was observed between 1921 and 1960 and from 1981 onwards, whereas an increase was found during the decades between 1961 and 1980. Overall, the time slot between 1901 and 1920 was found to be most productive as 64 species of the genus were proposed during this period, followed by 1881–1900 (47); 1921–1940 (36); 1961–1980 (33); 1981–2000 (20) and 2001–2020 (17). From the publication of the first species of fungi in 1853 to 1900, a total of 55 new species were reported; this number increased to 175 in the following century (1901–2000). Although this number is less (17) in the current century (Figure 4), it will be increased with the use of modern techniques of fungal identification.

These rust fungi cause distorted plant growth (witches’ broom) and defoliation, twig dieback, elongated and twisted stem-like symptoms in the infected host, which increase with the progress of the disease. These leaves of diseased hosts can turn yellow and fall prematurely, which can affect their strength. The death of plants is also observed in extreme cases of rust diseases [1,11]. The removal and destruction of infected plant parts can be used to manage this rust disease. However, the use of fungicides and biocontrol agents is also recommended in disease control. Dusting or foliar spray of sulfur-based fungicide (0.05%) is found to be effective in minimizing the disease [153].

A high-rank classification study performed by Aime and McTaggart [23] based on their 16 years of sampling included ca. 80% of accepted genera including type species wherever possible, and three DNA loci to resolve the deeper nodes of the rust fungus tree of life. Based on this and other studies [23,154,155], out of the total species currently placed in *Ravenelia*, many are now placed in other genera like *Cephalotelium*, *Cystingophora*, *Kernkampella*, *Lipocystis*, *Neoravenelia*, *Raveneliopsis*, etc. A number of *Ravenelia* spp. have now been treated as basionyms for the new fungal group where they are placed. The species of *Ravenelia*, viz. *R. appendiculata*, *R. brevispora*, *R. breyniae*, *R. breyniae*, *R. breyniae-patentis*, *R. coimbatorica*, *R. Emblicae*, *R. kirganeliae* and *R. phyllanthi*, have now been treated as basionyms of different species of the rust genus *Kernkampella* [155,156]. Similar observations were made of many various other species of *Ravenelia*. Although the traditional and DNA-based methods have their own utility and importance, both presented a unique transformation in the study of fungi. While basic or traditional methods are very popular in use due to their low cost and ease of use, the modern methods are now proved fruitful in identifying different fungi [157]. An outline of fungi presented by Wijayawardene et al. [158,159] considering both morphological and molecular techniques highlighted the importance of both techniques.

## 6. Conclusions and Future Perspectives

Around 248 species of *Ravenelia* have been identified on different plant hosts all around the world, of which approximately 19% (48) species are represented with molecular data. There are several possible factors behind this, including the fact that isolating DNA directly from naturally infected host samples and then achieving its sequencing is difficult. Additionally, the non-availability of resources, including modern facilities and funding, might be one of the major factors behind the reduced availability of molecular data. The molecular studies carried out by Aime and McTaggart [23], Wood and Aime [154] and Ebinghaus et al. [43] revealed the polyphyletic nature of the species of *Ravenelia* and raised the requirement of their thoughtful re-evaluation for how to reassign these species into monophyletic genera. More DNA-based analyses of other species within the genus are required for a better understanding of their taxonomic placement. The number of species that have now been described based on morphology needs to be recollected to supplement molecular data for accurate and precise identification and phylogenetic placement. However, the availability of well-trained mycologists with good expertise in traditional fungal classification, molecular systematics and bioinformatics/genomics is one of the major limitations that need to be resolved so that mycology and mycologists can be conserved and preserved.

## Figures and Tables

**Table 1:** GenBank and voucher/culture collection accession numbers of *Ravenelia* species used in the phylogenetic study.

Taxa	Isolate Name	GenBank Accession No.		References
		ITS	LSU	
*Ravenelia acaciae-arabicae*	PREM61853	N/A	MN072675	[2]
*Ravenelia acaciae-nigrescentis*	KR-M-0006413	N/A	MN072686	[2]
*Ravenelia acaciae-pennatulae*	U115	N/A	MG907213	[41]
*Ravenelia acaciicola*	PREM61861	N/A	MN072683	[2]
*Ravenelia arizonica*	BPI 878098	N/A	MW147032	[23]
*Ravenelia cebil*	BPI841029	N/A	MN072649	[2]
*Ravenelia cohniana*	BPI841185	N/A	MG954487	[38]
*Ravenelia dichrostachydis*	KR-M-0006448	N/A	MN072690	[2]
*Ravenelia doidgeae*	PREM60992	N/A	MN072672	[2]
*Ravenelia dumeti*	PREM61877	N/A	MN072681	[2]
*Ravenelia echinata* var. *ectypa*	BPI841034	N/A	DQ323925	[37]
*Ravenelia elephantorhizae*	KR-M-0006449	MN072702	MN072702	[2]
*Ravenelia escharoides*	WM3405	N/A	MG954480	[38]
*Ravenelia escharoides*	PREM61028	N/A	MG945996	[38]
*Ravenelia glabra*	KR-M-0006450	N/A	MN072691	[2]
*Ravenelia halsei*	PREM61855	N/A	MG954484	[38]
*Ravenelia havanensis*	BPI871922	N/A	MN275524	[2]
*Ravenelia hermosa*	BPI1107966	N/A	MN072657	[2]
*Ravenelia hernandezii*	BPI872308	N/A	MG954488	[38]
*Ravenelia hieronymi*	BPI841165	N/A	MN072651	[2]
*Ravenelia holwayi*	BPI871145	N/A	MN072656	[2]
*Ravenelia indigoferae*	PREM61061	N/A	MN072664	[2]
*Ravenelia inornata*	KR-M-0006613	N/A	MN072666	[2]
*Ravenelia lonchocarpi*	BPI 863495	N/A	MW147034	[23]
*Ravenelia macowaniana*	PREM61218	MG945979	N/A	[3]
*Ravenelia macowaniana*	PREM61218	N/A	MG946011	[38]
*Ravenelia macrocarpa*	BPI841195	N/A	DQ323926	[37]
*Ravenelia mainsiana*	BPI871923	N/A	MN275525	[2]
*Ravenelia mesilliana*	KR-M-0006424	N/A	MN072693	[2]
*Ravenelia mimosae-sensitivae*	BPI841052	N/A	MN072650	[2]
*Ravenelia modesta*	PREM61884	N/A	MN072688	[2]
*Ravenelia modjadji*	PREM61023	N/A	MN072667	[2]
*Ravenelia molopa*	PREM61879	N/A	NG_068900	[2]
*Ravenelia moloto*	KR-M-0006445	N/A	MN072697	[2]
*Ravenelia neocaledoniensis*	BRIP56907	N/A	KJ862347	[42]
*Ravenelia ornata*	KR-M-0006447	N/A	MN072687	[2]
*Ravenelia pienaarii*	KR-M-0006442	N/A	MN072699	[2]
*Ravenelia piepenbringiae*	MP5157	N/A	MG954489	[38]
*Ravenelia platensis*	BPI841204	N/A	MN072652	[2]
*Ravenelia pretoriensis*	PREM61021	N/A	MN072665	[2]
*Ravenelia sessilis*	BRIP 59688	N/A	MW147035	[23]
*Ravenelia spinifera*	PREM 61895	N/A	NG_068903	[2]
*Ravenelia stictica*	PREM60784	N/A	MN072663	[2]
*Ravenelia tephrosiae*	PREM61895	N/A	MN072698	[2]
*Ravenelia transvaalensis*	PREM61024	N/A	MN072669	[2]
*Ravenelia verrucata*	BPI 910281	KY764176	KY764176	Unpublished
*Ravenelia xanthophloeae*	PREM61000	MG945984	N/A	[3]
*Ravenelia xanthophloeae*	PREM61000	N/A	MG946016	[38]
*Kernkampella breyniae*	BRIP:56909	N/A	KJ862346	[42]
*Raveneliopsis spiralis*	UB 23929	N/A	OQ247917	[43]
*Lipocystis acaciae-pennatulae*	BPI 864189	N/A	MG907213	[41]

**Table 2:** Diversity, host range, and distribution of species of *Ravenelia*.

Sr. No.	Species	Host	Family	Locality	Reference
1.	*Ravenelia acaciae-arabicae* Mundk. & Thirum.	On phyllodes of *Acacia arabica*	*Fabaceae*	Karnataka (India)	[44]
2.	*Ravenelia acaciae-caesiae* Tyagi & S.S. Prasad	On *Acacia caesia*	*Fabaceae*	Rajasthan (India)	[45]
3.	*Ravenelia acaciae-concinnae* Mundk. & Thirum.	On phyllodes of *Acacia concinna*	*Fabaceae*	Karnataka (India)	[43]
4.	*Ravenelia acaciae-intsiae* B.V. Patil & Thirum.	On *Acacia intsia*	*Fabaceae*	Maharashtra (India)	[46]
5.	*Ravenelia acaciae-melliferae* Bacc.	On *Acacia mellifera*	*Fabaceae*	Eritrea, Ethiopia	[47]
6.	*Ravenelia acaciae-micranthae* Dietel	On *Acacia anisophylla, * *Acacia crassifolia*	*Fabaceae*	Mexico	[14]
7.	*Ravenelia acaciae-nigrescentis* Ritschel, Berndt & Oberw.	On phyllodes of *Acacia nigrescens*	*Fabaceae*	Namibia	[48]
8.	*Ravenelia acaciae-pennatulae* Dietel	On *Acacia pennatula, Acacia* sp. *Vachellia pennatula*	*Fabaceae*	Guatemala, Mexico	[14]
9.	*Ravenelia acaciae-senegalae* Sanwal	On *Acacia senegal*	*Fabaceae*	Delhi (India)	[48]
10.	*Ravenelia acaciae-sumae* Thirum. & Mundk.	On phyllodes of *Acacia suma*	*Fabaceae*	Karnataka (India)	[43]
11.	*Ravenelia acaciicola* Sanwal [as *‘acacicola’*]	On *Acacia senegal*	*Fabaceae*	Delhi (India)	[49]
12.	*Ravenelia aculeifera* Berk.	On leaves of *Megonemium enneaphyllum*	*Fabaceae*	Sri Lanka	[50]
13.	*Ravenelia affinis* P. Syd. & Syd.	On leaves of *Calliandra turbinata*	*Fabaceae*	Mato Grosso (Brazil)	[51]
14.	*Ravenelia ajmerensis* Sanwal	On *Acacia* sp.	*Fabaceae*	Rajasthan (India)	[49]
15.	*Ravenelia alamosensis* Cummins & J.W. Baxter	On *Pithecellobium tortum*	*Fabaceae*	Sinaloa (Mexico)	[52]
16.	*Ravenelia albiziae* Dietel [as ‘*albizziae*’]	On leaves of *Albizia*	*Fabaceae*	Eritrea	[18]
17.	*Ravenelia albiziicola* Cummins	On leaves of *Albizia harveyi*	*Fabaceae*	Malawi	[53]
18.	*Ravenelia aloes* R. Dubey & Akh.K. Pandey [as *‘aloës’*]	On leaves of *Aloe vera*	*Asphodelaceae*	Madhya Pradesh (India)	[54]
19.	*Ravenelia amazonica* Syd. & P. Syd.	On leaves of *Pithecellobium corymbosum*	*Fabaceae*	Rio Grande do Sul (Brazil)	[55]
20.	*Ravenelia amylis* Speg.	On leaves of *Acacia riparia*	*Fabaceae*	Paraguay	[56]
21.	*Ravenelia antiguana* Cummins	On *Cassia biflora*	*Fabaceae*	Guatemala	[57]
22.	*Ravenelia argentinensis* Speg.	On *Vachellia lutea*	*Fabaceae*	Argentina	[58]
23.	*Ravenelia arizonica* Ellis & Everh.	On living leaves of *Prosopis juliflora*	*Fabaceae*	Arizona	[59]
24.	*Ravenelia armata* Syd. & P. Syd.	On leaves of *Calliandra*	*Fabaceae*	Roraima (Brazil)	[47]
25.	*Ravenelia arthurii* Long [as *‘arthuri’*]	On leaves of *Cassia emarginata, Senna bicapsularis*	*Fabaceae,*	Cuba, Dominican Republic, Haiti, Jamaica, Puerto Rico (USA), Venezuela	[60]
26.	*Ravenelia atrocrustacea* Henn.	On living leaves of *Swartzia*	*Fabaceae*	Amazonas (Brazil)	[61]
27.	*Ravenelia aurea* Cummins & J.W. Baxter	On *Acacia pringlei*	*Fabaceae*	Oaxaca (Mexico)	[52]
28.	*Ravenelia australis* Dietel & Neger	On leaves and petioles of *Acacia cavenia*	*Fabaceae*	Biobío (Chile)	[62]
29.	*Ravenelia bahiensis* Henn.	On branches of *Mimosa remansoana*	*Fabaceae*	Bahia (Brazil)	[63,64]
30	*Ravenelia bajacalensis* Cummins & J.W. Baxter	On *Lysiloma candidum*	*Fabaceae*	Baja California Sur (Mexico)	[52]
31.	*Ravenelia bakeriana* Dietel	On leaves of *Lonchocarpus* sp.	*Fabaceae*	Pará (Brazil)	[53]
32.	*Ravenelia baumiana* Henn.	On leaves of *Cassia goratensis*	*Fabaceae*	Angola	[65]
33.	*Ravenelia bella* Cummins & J.W. Baxter	On *Cassia emarginata, * *Cassia atomaria*	*Fabaceae*	Mexico	[66]
34.	*Ravenelia bezerrae* Dianese, R.B. Medeiros & Furlan.	On leaves of *Enterolobium ellipticum*	*Fabaceae*	Brazília Distrito Federal (Brazil)	[67]
35.	*Ravenelia bifenestrata* Mains	On living leaves of *Pithecellobium platylobum*	*Fabaceae*	Veracruz (Mexico)	[68]
36.	*Ravenelia bizonata* Arthur & Holw.	On *Calliandra houstoni, Calliandra houstoniana* and *Calliandra* sp.	*Fabaceae*	Guatemala	[69]
37.	*Ravenelia brongniartiae* Dietel & Holw.	On leaves of *Brongniartia intermedia*	*Fabaceae*	Morelos (Mexico)	[70]
38	*Ravenelia burmanica* Thaung	On leaves of *Acacia concinna*	*Fabaceae*	Myanmar	[71]
39.	*Ravenelia candussioi* Ciccar.	On *Acacia laeta*	*Fabaceae*	Eritrea	[72]
40.	*Ravenelia cassiae-covesii* Long & Goodd.	On leaves of *Cassia covesii*	*Fabaceae*	Arizona	[73]
41.	*Ravenelia cassiicola* G.F. Atk. [as *‘cassiaecola’*]	On stems, leaves and pods of *Cassia nictitans*	*Fabaceae*	Alabama and Mississippi (US)	[74]
42.	*Ravenelia caulicola* Arthur	On leaves of *Cracca cinerea*	*Fabaceae*	Bahamas	[75]
43.	*Ravenelia cebil* Speg.	On living leaves of *Anadenanthera macrocarpa*, *Anadenanthera colubrina*	*Fabaceae*	Tucumán (Argentina), Brazil	[76]
44.	*Ravenelia cenostigmatis* Berndt & F.O. Freire	On leaves of *Cenostigma gardnerianum*	*Fabaceae*	Piauí (Brazil)	[77]
45.	*Ravenelia cerradensis* Rezende & Dianese	On leaves of *Chamaecrista claussenii* var. *cyclop-hylla*	*Fabaceae*	Mato Grosso (Brazil)	[36]
46.	*Ravenelia chacoensis* J.C. Lindq.	On *Prosopis nigra*	*Fabaceae*	Argentina	[78]
47.	*Ravenelia chapadensis* Rezende & Dianese	On leaves of *Chamaecrista decumbens*	*Fabaceae*	Goiás (Brazil)	[36]
48.	*Ravenelia clemensae* Syd.	On leaves of *Albizia procera*	*Fabaceae*	Philippines	[79]
49.	*Ravenelia cohniana* Henn.	On leaves of *Caesalpinia*	*Fabaceae*	Rio de Janeiro (Brazil)	[80]
50.	*Ravenelia comptula* Syd.	On phyllodes of *Acacia or Calliandra*	*Fabaceae*	Ecuador	[81]
51.	*Ravenelia concinna* Syd.	On living leaves of *Acacia polyphylla*, *Acacia riparia* and of *Acacia glomerosa*	*Fabaceae*	Venezuela	[82]
52.	*Ravenelia corbula* J.W. Baxter	On *Caesalpinia eriostachys*	*Fabaceae*	Sinaloa (Mexico)	[83]
53.	*Ravenelia corbuloides* J.F. Hennen & Cummins	On *Caesalpinia bracteosa*	*Fabaceae*	Bahia (Brazil)	[84]
54.	*Ravenelia costae* Salazar-Yepes & A.A. Carvalho	On leaves of *Pseudopiptadenia leptostachya*	*Fabaceae*	Rio de Janeiro (Brazil)	[85]
55.	*Ravenelia cumminsii* J.W. Baxter	On phyllodes of *Acacia willardiana*	*Fabaceae*	Sonora (Mexico)	[86]
56.	*Ravenelia decidua* (Peck) Holw.	On *Acacia greggi*	*Fabaceae*	Texas (US), Arizona	[87]
57.	*Ravenelia deformis* Tyagi & S.S. Prasad	On twigs of *Dichrostachys cinerea*	*Fabaceae*	Rajasthan (India)	[45]
58.	*Ravenelia densifera* J.F. Hennen & Cummins	On *Senna silvestris*	*Fabaceae*	São Paulo (Brazil)	[84]
59.	*Ravenelia dichrostachydis* Doidge	On leaves of *Dichrostachys glomerata*	*Fabaceae*	KwaZulu-Natal (South Africa)	[88]
60.	*Ravenelia dieteliana* Henn.	On leaves of *Calliandra macrocephala*	*Fabaceae*	Goiás (Brazil)	[89]
61.	*Ravenelia distans* Arthur & Holw.	On leaves *Calliandra* sp.	*Fabaceae*	Guatemala	[69]
62.	*Ravenelia doidgeae* M. Ebinghaus, Begerow & W. Maier	On *Senegalia polyacantha*	*Fabaceae*	South Africa	[2]
63.	*Ravenelia dumeti* M. Ebinghaus, W. Maier & Begerow	On leaves of *Senegalia brevispica*	*Fabaceae*	South Africa	[2]
64.	*Ravenelia dysocarpae* Long & Goodd.	On *Mimosa dysocarpa*	*Fabaceae*	Arizona	[90]
65.	*Ravenelia echinata* Lagerh. & Dietel	On leaves of *Calliandra*	*Fabaceae*	Ecuador	[18]
66.	*Ravenelia elephantorhizae* Doidge	On leaves of *Elephantorrhiza elephantina*	*Fabaceae*	Gauteng (South Africa)	[88]
67.	*Ravenelia emaensis* Rezende & Dianese	On leaves of *Anadenanthera*	*Fabaceae*	Goiás (Brazil)	[36]
68.	*Ravenelia eminens* J.F. Hennen & Cummins	On *Senna multijuga*	*Fabaceae*	Minas Gerais (Brazil)	[84]
69.	*Ravenelia entadae* Lagerh. & Dietel	On *Entada polystachya*	*Fabaceae*	Panama	[18]
70.	*Ravenelia epiphylla* (Schwein.) Dietel	On *Cracca virginiana, * *Galega virginiana, Tephrosia hispidula, Tephrosia lindheimeri, Tephrosia purpurea, Tephrosia spicata, Tephrosia talpa, Tephrosia tenella, Tephrosia virginiana*	*Fabaceae*	United States, Mexico	[18]
71.	*Ravenelia erythrinae* Gäum.	On *Erythrina velutina, * *Erythrina brucei*	*Fabaceae*	Ethiopia, Indonesia	[91]
72.	*Ravenelia escharoides* Syd. & P. Syd.	On phyllodes of *Acacia burkei*	*Fabaceae*	South Africa	[51]
73.	*Ravenelia esculenta* Naras. & Thirum.	Acacia arabica, Acacia eburnea	*Fabaceae*	Maharashtra (India)	[92]
74.	*Ravenelia evansii* Syd. & P. Syd.	On phyllodes of *Acacia robusta*	*Fabaceae*	Gauteng (South Africa)	[51]
75.	*Ravenelia expansa* Dietel & Holw.	On leaves of *Acacia tequilana*	*Fabaceae*	Jalisco (Mexico)	[70]
76.	*Ravenelia faceta* H.S. Jacks. & Holw.	On living leaves of *Cassia* sp.	*Fabaceae*	Rio de Janeiro (Brazil)	[93]
77.	*Ravenelia farlowiana* Dietel	On phyllodes of *Acacia anisophylla* and of *Acacia crassifolia*	*Fabaceae*	Mexico	[87]
78.	*Ravenelia fimbriata* Speg.	On living leaves of *Sesbania*	*Fabaceae*	São Paulo (Brazil)	[94]
79.	*Ravenelia floridana* Cummins & J.W. Baxter	On *Pithecellobium unguis-cati*	*Fabaceae*	Florida	[52]
80.	*Ravenelia formosana* P. Syd. & Syd.	On leaves of *Acacia farnesiana*	*Fabaceae*	Taiwan	[47]
81.	*Ravenelia fragrans* Long	On leaves of *Mimosa fragrans*	*Fabaceae*	Texas (US)	[13]
82.	*Ravenelia geminipora* J.F. Hennen & Cummins	On *Plathymenia reticulata*	*Fabaceae*	Minas Gerais (Brazil)	[84]
83.	*Ravenelia glabra* Kalchbr. & Cooke	On phyllodes of *Acacia horrida*	*Fabaceae*	South Africa	[95]
84.	*Ravenelia glanduliformis* Berk. & M.A. Curtis	On leaves of *Tephrosia hispida*	*Fabaceae*	South Carolina and Georgia	[96]
85.	*Ravenelia gooddingii* Long	On leaves of *Acacia suffrutescens*	*Fabaceae*	Arizona	[73]
86.	*Ravenelia goyazensis* Henn.	On leaves of *Andira pisonis*	*Fabaceae*	Goiás (Brazil)	[89]
87.	*Ravenelia gracilis* Arthur	On leaves of *Mimosaceae*	*Fabaceae*	San Luis Potosí (Mexico)	[97]
88.	*Ravenelia guyanensis* J.R. Hern.	On leaves of *Chamaecrista adiantifolia*	*Fabaceae*	Guyana	[98]
89.	*Ravenelia halsei* Doidge	On leaf petioles of *Acacia ataxacantha*	*Fabaceae*	KwaZulu-Natal (South Africa)	[99]
90.	*Ravenelia hansfordii* Cummins	On phyllodes of *Acacia*	*Fabaceae*	Uganda	[100]
91.	*Ravenelia hassleri* Speg.	On leaves of *Enterolobium timbouva*	*Fabaceae*	Paraguay	[101]
92.	*Ravenelia havanensis* Arthur	On leaves of *Enterolobium cyclocarpum*	*Fabaceae*	Cuba	[102]
93.	*Ravenelia henningsiana* Dietel	On *Piptadenia* sp.	*Fabaceae*	Brazil	[14]
94.	*Ravenelia hermosa* Cummins & J.W. Baxter	On *Leucaena palmeri*	*Fabaceae*	Sonora (Mexico)	[52]
95.	*Ravenelia hernandezii* Ebinghaus & Begerow	On *Senegalia tenuifolia*	*Fabaceae*	Costa Rica	[38]
96.	*Ravenelia hieronymi* Speg.	On living branches of *Acacia cavenia*	*Fabaceae*	Córdoba (Spain)	[103]
97.	*Ravenelia hobsonii* Cooke [as *‘hobsoni’*]	On *Pongamia pinnata*	*Fabaceae*	Bangladesh, India, Japan, Sri Lanka, Taiwan,	[19]
98.	*Ravenelia hoffmannseggiae* Long [as *‘hoffmanseggiae’*]	On leaves of *Hoffmannseggia oxycarpa*	*Fabaceae*	Texas (US)	[104]
99.	*Ravenelia holwayi* Dietel	On leaves of *Prosopis juliflora*	*Fabaceae*	California	[18]
100.	*Ravenelia humphreyana* Henn.	On leaves of *Cassia*	*Fabaceae*	Jamaica	[105]
101.	*Ravenelia idonea* H.S. Jacks. & Holw.	On living leaves of *Acacia riparia*	*Fabaceae*	São Paulo (Brazil)	[93]
102.	*Ravenelia igualica* Arthur	On leaves of *Acacia filiculoides*	*Fabaceae*	Guerrero (Mexico)	[75]
103.	*Ravenelia indica* Berk.	On leaves of *Abrus*	*Fabaceae*	Jharkhand (India)	[17]
104.	*Ravenelia indigoferae* Tranzschel ex Dietel	On leaves of *Indigofera palmeri*	*Fabaceae*	Jalisco (Mexico)	[87]
105.	*Ravenelia indigoferae-scabridae* F.L. Tai	On *Indigofera scabrida*	*Fabaceae*	Yunnan	[106]
106.	*Ravenelia indissimilis* F. Kern, Thurst. & Whetzel	On living leaves of *Mimosa arenosa*	*Fabaceae*	Venezuela	[107]
107.	*Ravenelia inornata* (Kalchbr.) Dietel	On *Acacia horrida, Acacia karroo, Vachellia karroo, Vachellia* sp.	*Fabaceae*	South Africa	[18]
108.	*Ravenelia inquirenda* Arthur & Holw.	On *Acacia bursaria*	*Fabaceae*	Guatemala	[69]
109.	*Ravenelia irregularis* Arthur	On leaves of *Cracca macrantha*	*Fabaceae*	Jalisco (Mexico)	[77]
110.	*Ravenelia japonica* Dietel & P. Syd.	On leaves of *Albizia julibrissi*	*Fabaceae*	Honshu (Japan)	[62]
111.	*Ravenelia juruensis* Syd. & P. Syd.	On leaves of *Pithecellobium glomeratum*	*Fabaceae*	Amazonas	[55]
112.	*Ravenelia karadensis* P.B. Chavan & U.V. Kulk.	On leaves of *Phyllanthus lawii*	*Phyllanthaceae*	Maharashtra (India)	[108]
113.	*Ravenelia laevioides* Arthur & Cummins	*Indigofera emarginella, Indigofera nigrescens, Indigofera* sp.	*Fabaceae*	Philippines, Uganda	[109]
114.	*Ravenelia laevis* Dietel & Holw.	On leaves of *Indigofera*	*Fabaceae*	México Distrito Federal and Jalisco (Mexico)	[70]
115.	*Ravenelia lagerheimiana* Dietel	On leaves of *Calliandra*	*Fabaceae*	Ecuador	[18]
116.	*Ravenelia lata* J.F. Hennen & Cummins	On *Acacia glomerosa*	*Fabaceae*	Minas Gerais (Brazil)	[84]
117.	*Ravenelia leonensis* Syd.	On leaves of *Albizia adianthifolia*	*Fabaceae*	Sierra Leone	[110]
118.	*Ravenelia le-testui* Maubl.	On *Cassia* sp.	*Fabaceae*	Mozambique	[111]
119.	*Ravenelia leucaenae* Long	On leaves of *Leucaena diversifolia*	*Fabaceae*	Oaxaca (Mexico)	[13]
120.	*Ravenelia leucaenae-microphyllae* Dietel	On *Acacia angustissima, * *Acacia* sp.	*Fabaceae*	Brazil, Guatemala	[14]
121.	*Ravenelia linda* Cummins & J.W. Baxter	On *Calliandra tapirorum*	*Fabaceae*	Honduras	[52]
122.	*Ravenelia lindquistii* J.F. Hennen & Cummins	On *Acacia praecox*	*Fabaceae*	Salta (Argentina)	[52]
123.	*Ravenelia lonchocarpi* Lagerh. & Dietel	On living leaves of *Lonchocarpus campestris*	*Fabaceae*	Minas Gerais (Brazil)	[18]
124.	*Ravenelia lonchocarpicola* Speg.	On leaves of *Lonchocarpus nitidus*	*Fabaceae*	Guatemala	[58]
125.	*Ravenelia longiana* Syd. & P. Syd.	On living leaves of *Cassia roemeriana*	*Fabaceae*	Texas (US)	[112]
126.	*Ravenelia lysilomae* Arthur	On leaves of *Lysiloma tergemina*	*Fabaceae*	Guerrero (Mexico)	[97]
127.	*Ravenelia macowaniana* Pazschke	On *Acacia horrida*,* Acacia karroo*,* Acacia seyal*,* Vachellia karroo*,* Vachellia* sp.	*Fabaceae*	South Africa	[18]
128.	*Ravenelia macrocapitula* F.L. Tai	On *Indigofera hancockii*	*Fabaceae*	Yunnan	[106]
129.	*Ravenelia macrocarpa* Syd. & P. Syd.	On living leaves of *Cassia bicapsularis*	*Fabaceae*	Brazil	[113]
130.	*Ravenelia macrocystis* Berk. & Broome	On stems and leaf of *Cassia tora*	*Fabaceae*	Sri Lanka	[50]
131.	*Ravenelia magnispina* Berndt	On leaves of *Cassia robiniifolia*	*Fabaceae*	Ecuador	[114]
132.	*Ravenelia mainsiana* Arthur & Holw.	On *Mimosa alba*,* Mimosa albida*,* Mimosa manzanilloana*,* Mimosa panamensis*,* Mimosa* sp.	*Fabaceae*	Colombia, Costa Rica Guatemala, Mexico	[69]
133.	*Ravenelia maranguensis* (Henn.) Cummins	On *Indigofera arrecta*, *Indigofera* sp.	*Fabaceae*	Tanzania, Malawi, Uganda, Ethiopia	[99]
134.	*Ravenelia mere* Cummins	On *Lonchocarpus michelianus* and *L. rugosus*	*Fabaceae*	Guatemala	[115]
135.	*Ravenelia mesilliana* Ellis & Barthol.	On leaves of *Cassia bauhinioides*	*Fabaceae*	New Mexico	[116]
136.	*Ravenelia mexicana* Tranzschel ex Dietel	On leaves of *Calliandra grandiflora*	*Fabaceae*	Jalisco (Mexico)	[87]
137.	*Ravenelia mexicensis* J.F. Hennen & Cummins	On *Leucaena macrocarpa*	*Fabaceae*	Jalisco (Mexico)	[84]
138.	*Ravenelia microcephala* Durrieu	On phyllodes of *Acacia concinna*	*Fabaceae*	Nepal	[117]
139.	*Ravenelia microcystis* Pazschke	On living leaves of *Cassia*	*Fabaceae*	Santa Catarina (Brazil)	[18]
140.	*Ravenelia microspora* Dietel	On leaves of *Cassia*	*Fabaceae*	São Paulo (Brazil)	[53]
141.	*Ravenelia millettiae* Hirats. f. & Hashioka	On living leaves of *Millettia reticulata*	*Fabaceae*	Taiwan	[118]
142.	*Ravenelia mimosae-albidae* Dietel	On *Mimosa albida, Mimosa casta*	*Fabaceae*	Antigua and Barbuda, Costa Rica, Guatemala, Peru, Dominica, Trinidad and Tobago, Venezuela	[14]
143.	*Ravenelia mimosae-coeruleae* Dietel	On *Mimosa caerulea*	*Fabaceae*	New Zealand	[14]
144.	*Ravenelia mimosae-himalayanae* S. Ahmad	On leaves of *Mimosa himalayana*	*Fabaceae*	Pakistan	[119]
145.	*Ravenelia mimosae-pudicae* F. Kern, Thurst. & Whetzel	On living leaves of *Mimosa pudica*	*Fabaceae*	Colombia	[120]
146.	*Ravenelia mimosae-sensitivae* Henn.	On leaves of *Mimosa sensitiva*	*Fabaceae*	Tucumán (Argentina)	[80]
147.	*Ravenelia mimosicola* Arthur	On leaves of *Mimosa stipitata*	*Fabaceae*	Guerrero (Mexico)	[75]
148.	*Ravenelia mineirosensis* Rezende & Dianese	On leaves of *Anadenanthera colubrina*	*Fabaceae*	Goiás (Brazil)	[36]
149.	*Ravenelia minima* Cooke	On leaves of *Albizia fastigiata*	*Fabaceae*	KwaZulu-Natal (South Africa)	[121]
150.	*Ravenelia minuta* Syd. & P. Syd.	On leaves of *Pithecellobium*	*Fabaceae*	Amazonas	[55]
151.	*Ravenelia mirandensis* F. Kern & Thurst.	On *Cassia tora*	*Fabaceae*	Venezuela	[122]
152.	*Ravenelia mitis* Syd. & P. Syd.	On leaves of *Tephrosia purpurea*	*Fabaceae*	Himalaya (India)	[123]
153.	*Ravenelia mitteri* Syd.	On living leaves of *Indigofera leptostachya*	*Fabaceae*	Uttarakhand (India)	[124]
154.	*Ravenelia modesta* Doidge	On phyllodes of *Acacia stolonifera*	*Fabaceae*	Gauteng (South Africa)	[99]
155.	*Ravenelia modjadji* M. Ebinghaus, W. Maier & Begerow	On *Senegalia polyacantha*	*Fabaceae*	South Africa	[2]
156.	*Ravenelia molopa* M. Ebinghaus, W. Maier & Begerow	On leaves of *Senegalia galpinii*	*Fabaceae*	-	[2]
157.	*Ravenelia moloto* W. Maier, M. Ebinghaus & Begerow	On *Senegalia* sp.	*Fabaceae*	South Africa	[2]
158.	*Ravenelia monosticha* Speg.	On leaves of *Acacia bonariensis*	*Fabaceae*	Paraguay	[56]
159.	*Ravenelia multispinosa* Cummins & J.W. Baxter	On *Pithecellobium tortum*	*Fabaceae*	Sonora (Mexico)	[52]
160.	*Ravenelia munduleae* Henn.	On *Mundulea sericea*	*Fabaceae*	South Africa	[125]
161.	*Ravenelia natalensis* Syd., P. Syd. & Pole-Evans	On phyllodes of *Acacia hirtella*	*Fabaceae*	KwaZulu-Natal (South Africa)	[51]
162.	*Ravenelia neocaledoniensis* B. Huguenin	On branches, petioles and leaves of *Acacia farnesiana*	*Fabaceae*	New Caledonia	[126]
163.	*Ravenelia odoratissimae* Tyagi & S.S. Prasad	On pinnules of *Albizia odoratissima*	*Fabaceae*	Rajasthan (India)	[45]
164.	*Ravenelia oligotheles* Speg.	On living leaves of *Enterolobium timbouva*	*Fabaceae*	Formosa (China)	[76]
165.	*Ravenelia omanensis* Gjaerum & D.A. Reid	On leaves of *Caesalpinia* cfr. *erianthera*	*Fabaceae*	Oman	[127]
166.	*Ravenelia ornamentalis* (Kalchbr.) Dietel	On *Acacia farnesiana*	*Fabaceae*	South Africa	[14]
167.	*Ravenelia ornata* Syd. & P. Syd.	On leaves of *Abrus pulchellus*	*Fabaceae*	Uttarakhand (India)	[128]
168.	*Ravenelia palenquensis* Berndt	On leaves of *Pithecellobium macradenium*	*Fabaceae*	Ecuador	[114]
169.	*Ravenelia papillosa* Speg.	On living leaves of *Albizia julibrissin*	*Fabaceae*	Buenos Aires (Argentina)	[129]
170.	*Ravenelia parahybana* Viégas	*Caesalpinia sp., Lonchocarpus* sp.	*Fabaceae*	Brazil	[130]
171.	*Ravenelia parasnathii* Yadav	On *Acacia canescens*	*Fabaceae*	Bihar (India)	[131]
172.	*Ravenelia pazschkeana* Dietel	On leaves of species of *Mimosaceae* sub-family	*Fabaceae*	Rio de Janeiro (Brazil)	[132]
173.	*Ravenelia peglerae* Doidge	On phyllodes of *Acacia caffra*	*Fabaceae*	South Africa	[88]
174.	*Ravenelia pennatae* Durrieu	On phyllodes of *Acacia pennata*	*Fabaceae*	Nepal	[117]
175.	*Ravenelia pernigra* J.F. Hennen & Cummins	On *Cratylia argentea*	*Fabaceae*	Mato Grosso do Sul (Brazil)	[84]
176.	*Ravenelia pienaarii* Doidge	On phyllodes of *Acacia caffra*	*Fabaceae*	Gauteng (South Africa)	[88]
177.	*Ravenelia piepenbringiae* Ebinghaus & Begerow	On *Senegalia hayesii*	*Fabaceae*	Panama	[38]
178.	*Ravenelia pileolarioides* Syd. & P. Syd.	On leaves of *Pithecellobium*	*Fabaceae*	Ceará (Brazil)	[55]
179.	*Ravenelia piscidiae* Long	On leaves of *Piscidia erythrina*	*Fabaceae*	Florida	[133]
180.	*Ravenelia pithecellobii* Arthur [as *‘pithecolobii’*]	On leaves of *Pithecellobium dulce*	*Fabaceae*	Jalisco (Mexico)	[97]
181.	*Ravenelia platensis* Speg.	On young branches, petioles and leaves of *Erythrina crista-galli*	*Fabaceae*	Buenos Aires, Jujuy and Chaco (Argentina)	[129]
182.	*Ravenelia portoricensis* Arthur	On leaves of *Cassia emarginata*	*Fabaceae*	Puerto Rico (USA)	[134]
183.	*Ravenelia pretoriensis* Syd. & P. Syd.	On phyllodes of *Acacia pennata*	*Fabaceae*	Gauteng (South Africa)	[51]
184.	*Ravenelia pringlei* Cummins	On *Acacia greggii*	*Fabaceae*	Sonora (Mexico)	[135]
185.	*Ravenelia prosopidicola* J.C. Lindq.	On *Prosopis talpataco*	*Fabaceae*	Argentina	[136]
186.	*Ravenelia prosopidis* Long	On leaves of *Prosopis juliflora*	*Fabaceae*	Texas (US)	[104]
187.	*Ravenelia pulcherrima* Arthur	On leaves of *Poinciana pulcherrima*	*Fabaceae*	Morelos (Mexico)	[97]
188.	*Ravenelia pygmaea* Lagerh. & Dietel	On leaves of *Phyllanthus*	*Phyllanthaceae*	Ecuador	[18]
189.	*Ravenelia rata* H.S. Jacks. & Holw.	On living leaves of *Acacia pedicellata*	*Fabaceae*	Rio de Janeiro (Brazil)	[93]
190.	*Ravenelia reticulatae* Long	On leaves of *Calliandra reticulata*	*Fabaceae*	Arizona	[137]
191.	*Ravenelia rioensis* J.F. Hennen & Cummins	On *Acacia* sp.	*Fabaceae*	Brazil	[84]
192.	*Ravenelia roemerianae* Long	On leaves of *Acacia roemeriana*	*Fabaceae*	Texas (US)	[104]
193.	*Ravenelia rubra* J.W. Baxter	On leaves of *Brogniartia*	*Fabaceae*	Sinaloa (Mexico)	[60]
194.	*Ravenelia santos-costae* Dianese, A.G. Medeiros, L.T.P. Santos & A.C. Dianese	On leaves of *Calliandra dysantha*	*Fabaceae*	Brazília Distrito Federal (Brazil)	[67]
195.	*Ravenelia sarmientoi* J.C. Lindq.	On leaves of *Bulnesia sarmientoi*	*Zygophyllaceae*	Argentina	[138]
196.	*Ravenelia satarensis* P.B. Chavan & U.V. Kulk.	On pods of *Caesalpinia sepiaria*	*Fabaceae*	Maharashtra (India)	[108]
197.	*Ravenelia sayeedii* M.A. Salam & Ramachar	On leaves of *Sophora glauca*	*Fabaceae*	India	[139]
198.	*Ravenelia schroeteriana* Henn. [as *‘schröteriana’*]	On leaves of *Indigofera*	*Fabaceae*	Salta (Argentina)	[80]
199.	*Ravenelia schweinfurthii* Syd. & P. Syd.	On living leaves of *Entada sudanica*	*Fabaceae*	Sudan	[113]
200.	*Ravenelia scopulata* Cummins & J.W. Baxter	On *Acacia greggii*	*Fabaceae*	Mexico	[140]
201.	*Ravenelia sensitiva* Speg.	On *Mimosa sensitiva*	*Fabaceae*	Argentina	[58]
202.	*Ravenelia septata* J.F. Hennen & Cummins	On *Mimosa* sp.	*Fabaceae*	Minas Gerais (Brazil)	[84]
203.	*Ravenelia sessilis* Berk.	On leaves of *Gleditsia*	*Fabaceae*	Sri Lanka	[50]
204.	*Ravenelia siderocarpi* Long	On leaves of *Siderocarpos flexicaulis*	*Fabaceae*	Texas (US)	[104]
205.	*Ravenelia similis* (Long) Arthur	On *Brongniartia podalyrioides, Brongniartia nudiflora*	*Fabaceae*	Mexico	[97]
206.	*Ravenelia simplex* Dietel	On leaves of *Piptadenia communis*	*Fabaceae*	Rio de Janeiro (Brazil)	[132]
207.	*Ravenelia sololensis* Arthur & Holw.	On leaves of *Lysiloma acapulcensis*	*Fabaceae*	Guatemala	[69]
208.	*Ravenelia sonorensis* Cummins	On *Acacia californica*	*Fabaceae*	Mexico	[141]
209.	*Ravenelia spegazziniana* J.C. Lindq.	On *Acacia aroma*	*Fabaceae*	Argentina	[78]
210.	*Ravenelia spicigerae* B.V. Patil & Thirum.	On *Prosopis spicigera*	*Fabaceae*	Maharashtra (India)	[46]
211.	*Ravenelia spinifera* W. Maier, M. Ebinghaus & Begerow	*Senegalia mellifera*	*Fabaceae*	South Africa	[2]
212.	*Ravenelia spinulosa* Dietel & Holw.	On leaves of *Cassia multiflora*	*Fabaceae*	Oaxaca (Mexico)	[142]
213.	*Ravenelia spiralis* J.F. Hennen & Cummins	On *Cenostigma*	*Fabaceae*	Goiás (Brazil)	[84]
214.	*Ravenelia stevensii* Arthur	On leaves of *Acacia riparia*	*Fabaceae*	Puerto Rico (USA)	[143]
215.	*Ravenelia stictica* Berk. & Broome	On leaves of *Pongamia glabra*	*Fabaceae*	Sri Lanka	[50]
216.	*Ravenelia striatispora* Cummins & J.W. Baxter	On *Pithecellobium mexicanum*	*Fabaceae*	Sinaloa (Mexico)	[52]
217.	*Ravenelia stuhlmannii* Henn. [as *‘stuhlmanni’*]	On leaves of *Cassia petersiana*	*Fabaceae*	Tanzania	[144]
218.	*Ravenelia subtortuosa* Long	On leaves of *Acacia suffrutescens*	*Fabaceae*	Texas (US)	[73]
219.	*Ravenelia sumatii* S.D. Patil & Date	On leaves of *Acacia intsia*	*Fabaceae*	Maharashtra (India)	[140]
220.	*Ravenelia sydowiana* Rick	On *Lonchocarpus leucanthus, Lonchocarpus* sp.	*Fabaceae*	Brazil	[145]
221.	*Ravenelia talpa* (Long) Arthur	On *Cracca talpa, Cracca macrantha, Tephrosia linearis, Tephrosia talpa*	*Fabaceae*	Mexico, Nigeria	[97]
222.	*Ravenelia tandonii* Syd.	On leaves and petioles of *Acacia catechu*	*Fabaceae*	Madhya Pradesh (India)	[146]
223.	*Ravenelia taslimii* Mundk.	On seeds of *Acacia modesta*	*Fabaceae*	Delhi (India)	[147]
224.	*Ravenelia tauaensis* Viégas	On *Lonchocarpus* sp.	*Fabaceae*	Brazil	[130]
225.	*Ravenelia tephrosiae* Kalchbr.	On *Tephrosia macropoda*	*Fabaceae*	KwaZulu-Natal (South Africa)	[18]
226.	*Ravenelia tephrosiicola* (Henn.) Hirats. f.	On leaves of *Tephrosia*	*Fabaceae*	Peru	[148]
227.	*Ravenelia tessellata* J.F. Hennen & Cummins	On *Parkia* sp.	*Fabaceae*	Pará (Brazil)	[84]
228.	*Ravenelia texensis* Ellis & Galloway	On species of *Acacia, Calliandra, Desmanthus, Schrankia, Morongia*	*Fabaceae*	Mexico, United States	[18]
229.	*Ravenelia theisseniana* Syd. & P. Syd.	On leaves of *Leguminosae* plants	*Fabaceae*	Rio Grande do Sul (Brazil)	[123]
230.	*Ravenelia thornberiana* Long	On leaves of *Acacia contricta*	*Fabaceae*	Texas (US)	[137]
231.	*Ravenelia tortuosa* J.F. Hennen & Cummins	On *Mimosoideae* plants	*Fabaceae*	Bahia (Brazil)	[84]
232.	*Ravenelia transvaalensis* Doidge	On leaves of *Acacia detinens*	*Fabaceae*	Gauteng (South Africa)	[99]
233.	*Ravenelia uleana* Henn.	On leaves of *Cassia*	*Fabaceae*	Goiás (Brazil)	[89]
234.	*Ravenelia urbaniana* Henn.	On leaves of *Cassia*	*Fabaceae*	Goiás (Brazil)	[149]
235.	*Ravenelia usambarae* Syd. & P. Syd.	On living leaves of *Cassia goratensis*	*Fabaceae*	Tanzania	[113]
236.	*Ravenelia venustula* Syd.	-	-	-	[150]
237.	*Ravenelia verrucata* Cummins & J.W. Baxter	On *Mimosa spirocarpa*	*Fabaceae*	Sinaloa (Mexico)	[52]
238.	*Ravenelia verrucosa* Cooke & Ellis	On leaves of *Leucania*	*Fabaceae*	Mexico	[151]
239	*Ravenelia versatilis* (Peck) Dietel	On different species of *Acacia*	*Fabaceae*	Brazil, Ibndia, Mexico, United States	[18]
240.	*Ravenelia victoria-rossetiae* Dianese, L.T.P. Santos, R.B. Medeiros & M. Sanchez [as *‘victoria*-*rossetii’*]	On leaves of *Mimosa radula* var. *imbricata*	*Fabaceae*	Brazília Distrito Federal (Brazil)	[67]
241.	*Ravenelia vilis* Syd. & P. Syd.	On leaves of *Piptadenia*	*Fabaceae*	Ceará (Brazil)	[54]
242.	*Ravenelia volkensii* Henn.	On stems of *Acacia*	*Fabaceae*	Tanzania	[18]
243.	*Ravenelia whetzelii* Arthur	On leaves of *Inga vera*	*Fabaceae*	Puerto Rico (USA)	[152]
244.	*Ravenelia wiehei* Cummins	On leaves of *Cassia singueana*	*Fabaceae*	Malawi	[53]
245.	*Ravenelia woodii* Pazschke	On leaves of *Leguminosae* plants	*Fabaceae*	South Africa	[18]
246.	*Ravenelia xanthophloeae* M. Ebinghaus, W. Maier & Begerow	On *Vachellia xanthophloea*	*Fabaceae*	South Africa	[3]
247.	*Ravenelia zeylanica* Dietel	On *Gleditsia* sp.	*Fabaceae*	Sri Lanka	[18]
248.	*Ravenelia zygiae* Syd.	On leaves of *Albizia zygia*	*Fabaceae*	Sierra Leone	[110]

## Data Availability

Not applicable.
